# Three decades of progress: evolution of outcomes and prognostic indicators in biliary atresia management

**DOI:** 10.1186/s12887-025-05848-6

**Published:** 2025-07-02

**Authors:** Senol Emre, Yalim Benibol, Ali Ekber Hakalmaz, Buse Karakurt, Ayse Kalyoncu Ucar, Nuray Kepil, Ömer Faruk Beşer, Sebuh Kuruğoğlu, Osman Faruk Senyuz

**Affiliations:** 1 Department of Pediatric Surgery, Istanbul University-Cerrahpasa, Cerrahpasa Faculty of Medicine, Yesilkoy, Istanbul, Turkey; 2 Department of Radiology, Istanbul University-Cerrahpasa, Cerrahpasa Faculty of Medicine, Yesilkoy, Istanbul, Turkey; 3 Department of Pathology, Istanbul University-Cerrahpasa, Cerrahpasa Faculty of Medicine, Yesilkoy, Istanbul, Turkey; 4 Department of Pediatric Gastroenterology, Istanbul University-Cerrahpasa, Cerrahpasa Faculty of Medicine, Yesilkoy, Istanbul, Turkey

**Keywords:** Biliary Atresia, Kasai portoenterostomy, APRI score, ALBI score, METAVIR score, Native liver survival, Prognostic factors

## Abstract

**Background:**

Biliary atresia (BA) remains a challenging condition with variable outcomes following Kasai portoenterostomy (KPE). This study evaluates the predictive value of three scoring systems (ALBI, APRI, and METAVIR) for prognostication and analyzes three decades of treatment outcomes at a single institution.

**Methods:**

Records of 96 BA patients who underwent KPE between 1990 and 2022 were retrospectively analyzed. Patients were stratified by preoperative ALBI (originally developed for HCC but applied here for its objective assessment of liver function), preoperative and postoperative APRI, and METAVIR scores. Treatment eras were divided into 1990–2000 (*n* = 18), 2001–2010 (*n* = 31), and 2011–2022 (*n* = 47). Clearance of jaundice was assessed at three months post-KPE, following established literature benchmarks. Kaplan-Meier curves and Log-Rank tests were used for survival analyses with a median follow-up of 5.1 years (range: 1–10 years).

**Results:**

The 10-year native liver survival (NLS) rate was 52.4%, with overall survival (OS) of 69.6%. Preoperative ALBI and APRI scores showed limited prognostic value (*p* = 0.12 and *p* = 0.17, respectively). However, postoperative APRI scores exceeding 1.12 at three months significantly predicted poor outcomes (*p* = 0.00094). METAVIR scores demonstrated poor correlation with long-term outcomes (*p* = 0.26). Analysis across treatment eras revealed significant improvement, with NLS increasing from 42.1 to 64.8% (*p* = 0.0029) and OS from 56.3 to 85.2% (*p* < 0.0001) between 1990 and 2000 and 2011–2022. Among the 52 patients with completed 10-year follow-up, the jaundice clearance rate at three months was 31.2%.

**Conclusions:**

While preoperative scoring systems showed limited predictive ability, postoperative APRI scores at three months emerged as a powerful predictor of long-term outcomes in BA patients. Our findings suggest that patients with three-month postoperative APRI scores > 1.12 should receive more intensive monitoring and earlier consideration for transplantation. The significant improvement in outcomes across three decades demonstrates the value of accumulated institutional experience and improved clinical management protocols in this challenging disease.

**Trial registration:**

Not applicable.

## Introduction

Biliary atresia remains a progressive liver disease that poses significant challenges in diagnosis and surgical treatment, making it difficult to achieve optimal outcomes. While overall survival is a primary concern in treatment, the optimal outcome is jaundice-free native liver survival. Biliary atresia is notably the most common indication for pediatric liver transplantation worldwide, underscoring the importance of effective prognostic tools. Numerous scoring systems have been developed to predict postoperative outcomes in patients undergoing the Kasai portoenterostomy (KPE). One such scoring system is the Albumin-Bilirubin (ALBI) score, initially described by Johnson et al. [1]. Previously utilized to evaluate liver function in patients with hepatocellular carcinoma, the ALBI score has also been reported as applicable in primary biliary cirrhosis [2]. Notably, the ALBI score does not include any subjective factors. Another scoring system is the Aspartate to Platelet Ratio Index (APRI), based solely on laboratory results [3, 4]. A prospective series conducted at King’s College reported that the APRI value at the time of Kasai portoenterostomy serves as a useful tool in assessing the severity of liver disease in biliary atresia cases [5]. Additionally, the METAVIR histopathological scoring system, which is used to evaluate the degree of liver fibrosis, is another valuable tool in this context [6, 7]. Other prognostic factors known to influence outcomes include:

1. The age at diagnosis must be less than 60 days [8]. For patients diagnosed between 60 and 90 days, success rates vary depending on the condition of the liver and the success of the surgical procedure.
2. The degree of hepatic fibrosis at the time of diagnosis and treatment [9].
3. The diameters of bile ducts at the porta hepatis [10].

This study aims to assess the relationship between these prognostic factors and surgical outcomes in children undergoing the Kasai procedure for biliary atresia at our center, as well as to evaluate single-center outcomes.

## Materials and methods

This study was conducted with the approval of the Local Clinical Research Ethics Committee. Records of patients who underwent the Kasai procedure for biliary atresia at our center between 1990 and 2022 were retrospectively analyzed.

### Patient selection and diagnostic approach

In cases referred to our clinic due to prolonged jaundice, the presence of direct hyperbilirubinemia and acholic stools was evaluated. A six-hour fasting ultrasonography was used as the initial diagnostic tool, alongside routine biochemical examinations and viral serology. Criteria for elective laparoscopy included liver parenchymal changes, particularly hyperechogenicity at the porta hepatis, coarsening of structure, reduced gallbladder size and compliance (postprandial), ascites, or components of the biliary atresia splenic malformation syndrome (BASM). Preoperative assessments included echocardiography, ophthalmologic examination to exclude posterior embryotoxon, and babygram imaging for Alagille syndrome investigation.

### Surgical procedure

All operations were performed by two surgeons. Laparoscopy was performed for diagnostic confirmation. Laparoscopic findings of parenchymal nodularity, coarsened structure, green-brown liver discoloration, hypoplastic gallbladder, and absence of intrahepatic bile ducts on cholangiography warranted laparotomy via a short left Chevron incision. In cases aged ≥ 90 days with advanced ascites, severe parenchymal disruption, pronounced nodularity, or extensive angiomatous formations, a tru-cut biopsy was taken, and the procedure was terminated to avoid complicating future liver transplantation [11]. Portoenterostomy was constructed according to the extended Kasai procedure with a 55 cm Roux limb to minimize cholangitis frequency. Liver wedge biopsies were obtained from the right anterior sector for histopathological examination.

### Postoperative management

Postoperative care included monitoring of weight gain, sclera color, and stool color. Clearance of jaundice was defined as total bilirubin levels < 2 mg/dL and passage of colored stools within three months. Liver function tests were recorded at two weeks, one, two, three, six months, and one year postoperatively. Patients with no complications were followed annually with ultrasound and laboratory tests. Complications such as gastrointestinal bleeding, massive splenomegaly, hypersplenism, hepatopulmonary syndrome, bile lakes, or frequent hospitalizations due to recurrent cholangitis led to liver transplant listing.

### Inclusion criteria and study parameters

Patients with complete preoperative, perioperative, and postoperative laboratory data, regular follow-up, and updated social security records were included. Data analyzed included age at surgery, congenital infections, preoperative and postoperative scoring parameters, and liver biopsy pathology reports.

### Scoring systems

#### ALBI score

Calculated as (log_1_₀ bilirubin×0.66) + (albumin×−0.085) Patient classification:

- Group 1 (low mortality): <-2.6.
- Group 2 (moderate mortality): -2.6 to -1.39.
- Group 3 (high mortality): > -1.39.

#### APRI score

Calculated as [(AST/ULN)/platelet count (10⁹/L)]×100 Patient classification:

- Group 1: < 0.43.
- Group 2: 0.43–0.67.
- Group 3: 0.67–1.12.
- Group 4: > 1.12.

#### METAVIR score

Liver fibrosis assessment:

- 0: No fibrosis observed.
- 1: Fibrosis extending to the portal areas.
- 2: Fibrosis extending to portal areas with rare bridging fibrosis.
- 3: Fibrosis with pronounced bridging and nodularity.
- 4: Cirrhosis.

### Statistical analysis

Statistical analyses were conducted using Python 3.9.2. Survival analyses included both native liver survival (NLS) and overall survival (OS) rates, which were evaluated using Kaplan-Meier curves and Log-Rank tests. For comparing outcomes across different treatment eras (1990–2000, *n* = 18; 2001–2010, *n* = 31; and 2011–2022, *n* = 47), Chi-square tests of independence were performed, with subsequent pairwise comparisons using Bonferroni-corrected *p*-values (significance level set at *p* < 0.0167). Effect sizes were calculated using Cramer’s V. Jaundice clearance and APRI score distribution graphs were generated to observe outcomes at three months postoperatively. Statistical significance was defined as *p* < 0.05 unless otherwise specified.

## Results

### Patient overview

Between 1990 and 2022, 143 infants with biliary atresia were treated at our center. After applying the inclusion criteria, 96 cases (67%) with complete prognostic data and long-term follow-up were included in the final analysis. Of the excluded patients, 27 cases had incomplete data or were lost to follow-up, 17 cases were identified as having advanced cirrhosis during diagnostic laparoscopy and directly listed for transplantation, and 3 patients died within six weeks postoperatively due to non-biliary atresia-related causes. The study cohort consisted of 42 males and 54 females, with a mean age at surgery of 57 days (range: 14–131 days). Table 1 shows the age distribution of patients at the time of Kasai portoenterostomy (KPE).

**Table 1:**
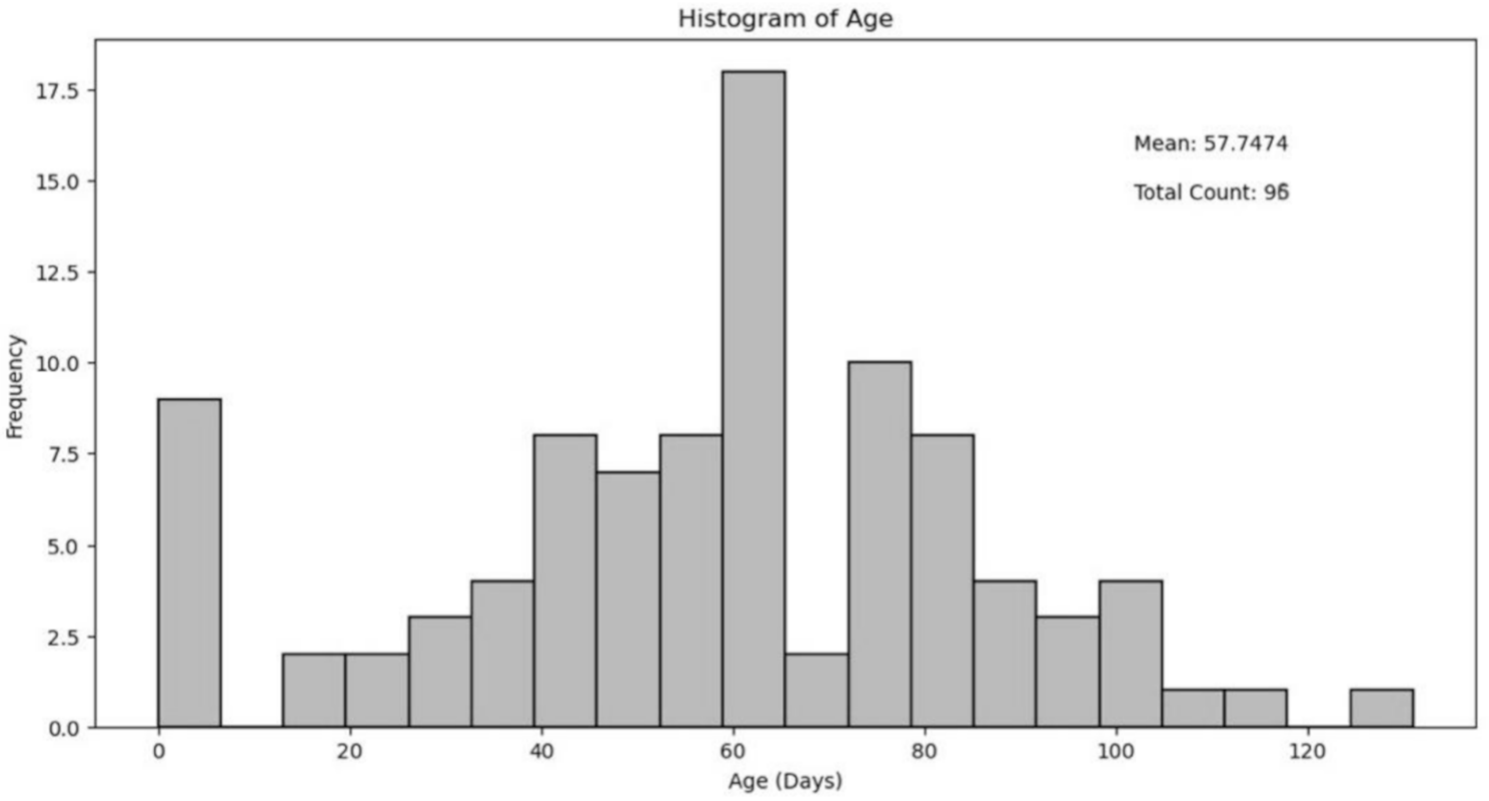
Age distribution of patients at the time of Kasai portoenterostomy (KPE). Histogram shows the frequency distribution of patient ages in days, with a mean age of 57 days (range: 14–131 days). The highest frequency was observed in the 60–65 days range (*n* = 18), with notable frequencies also in the 0–5 days (*n* = 9) and 70–80 days (*n* = 10) ranges. Total patient count: 96

### Jaundice clearance

Clearance of jaundice was assessed at the third postoperative month. Based on existing literature and clinical experience jaundice-clearance at 3-months post-operatively is a good early indicator of long-term success of Kasai portoenterostomy [12]. Clearance of jaundice, defined as total bilirubin levels < 2 mg/dL and passage of colored stools within three months postoperatively, was achieved in 30 cases (31.2%). The median APRI score for these jaundice-free cases was 0.60, indicating significant improvement compared to preoperative values.

### Survival analysis

The 10-year native liver survival (NLS) rate was 52.4%, while the overall survival rate was 69.6%. Of the 96 patients (Table 2):

- 53 (55.2%) continued living with their native liver.
- 17 (17.7%) underwent liver transplantation.
- 26 (27.1%) died following KPE.

Among the 17 transplanted cases:

- 6 patients under one year of age had a 100% survival rate post-transplant.
- 11 patients over one year of age underwent transplantation, with 3 deaths reported post-transplant.

Overall, 14 of 17 (82.4%) transplant recipients survived with the transplanted liver.

Of the 96 patients included in the study, 52 patients had completed 10 years of follow-up at the time of analysis and were included in the 10-year survival calculations. The remaining patients contributed to survival analysis until their last documented follow-up visit, with a median follow-up duration of 5,1 years (range: 1–10 years).

**Table 2:**
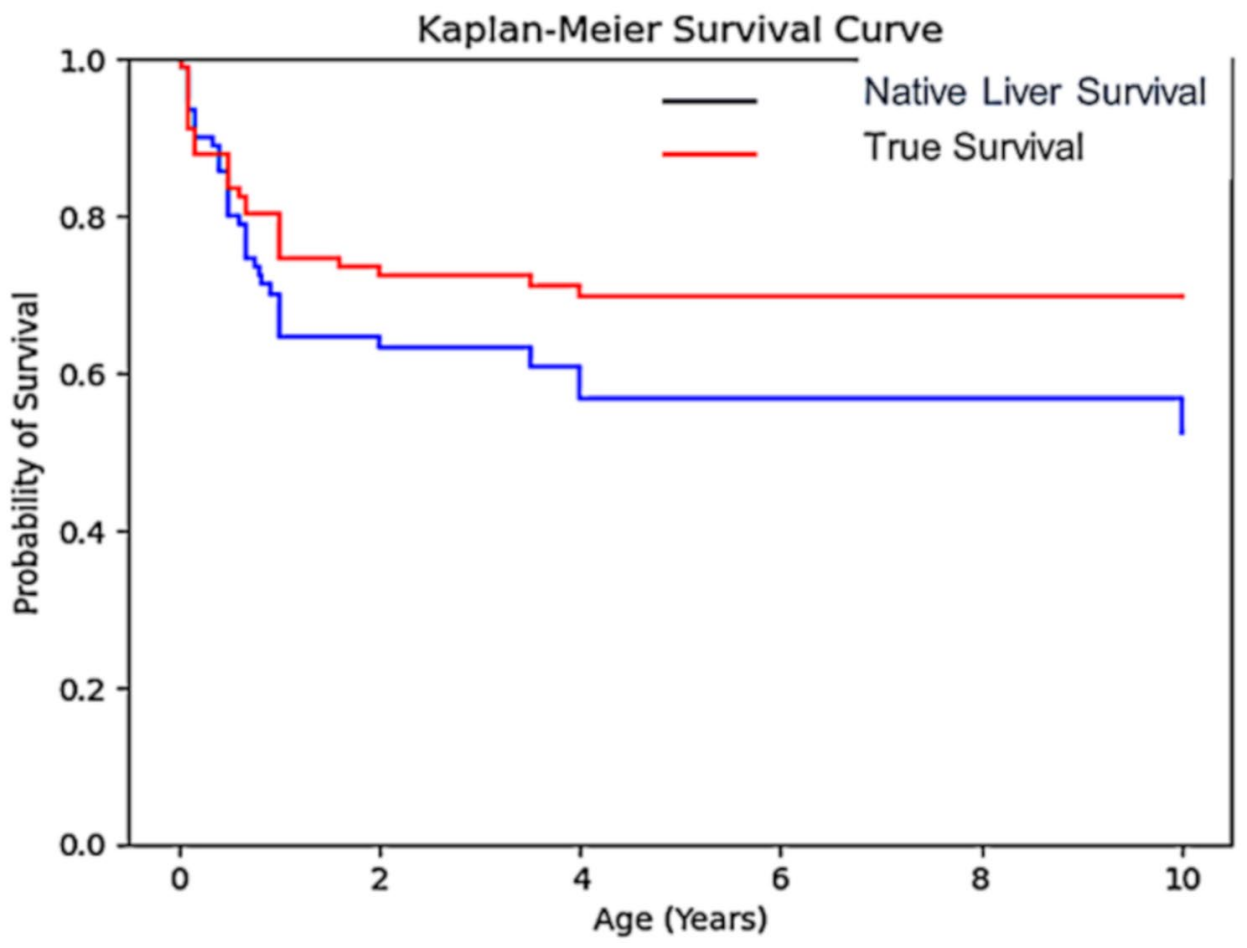
Survival rates overview. The true survival includes patients who underwent liver transplantation, representing the overall survival rate of the entire cohort regardless of whether patients maintained their native liver or received a transplant

### Era analysis

When analyzing outcomes by treatment era, we observed a progressive improvement in both native liver survival (NLS) and overall survival (OS) rates across three decades:

- 1990–2000 (*n* = 18): 10-year NLS rate of 42.1%, overall survival 56.3%.
- 2001–2010 (*n* = 31): 10-year NLS rate of 50.7%, overall survival 67.5%.
- 2011–2022 (*n* = 47): 10-year NLS rate of 64.8%, overall survival 85.2%.

Statistical analysis revealed significant differences across the three eras for both survival measures (Chi-square test, *p* < 0.05). The most substantial improvements were observed between the earliest (1990–2000) and most recent (2011–2022) eras, with statistically significant increases in both NLS (42.1% vs. 64.8%, *p* = 0.0029) and OS (56.3% vs. 85.2%, *p* < 0.0001). The intermediate era (2001–2010) showed transitional improvement, though differences between consecutive eras did not reach statistical significance for NLS rates (*p* > 0.0167).

### Age at operation

Survival analysis using Kaplan-Meier curves (Table 3) showed:

- Patients operated on under 30 days of age had lower 10-year NLS rates compared to other groups, though this difference was not statistically significant (*p* = 0.093).
- Patients operated on after 90 days of age had survival rates comparable to other groups (*p* = 0.70).

**Table 3:**
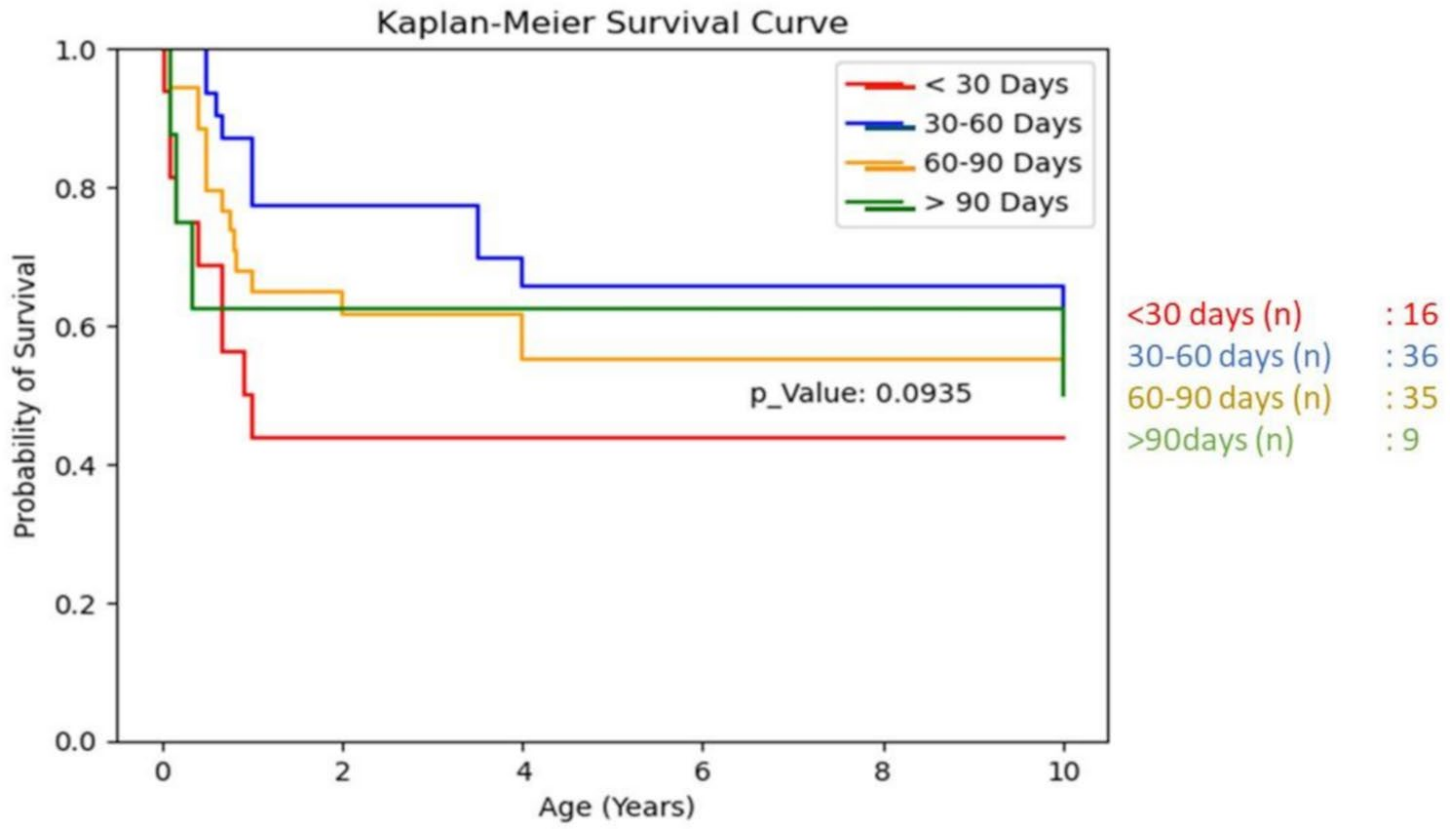
Age at operation and native liver survival rates. The data shows an unexpected finding of lower 10-year NLS rates in patients operated on before 30 days of age compared to other groups, though this difference was not statistically significant (*p* = 0.093)

### Prognostic scoring systems

#### ALBI scoring

No patients were classified as Group 1 (low mortality) based on preoperative ALBI scoring. The differences in native liver survival between Group 2 (moderate mortality) and Group 3 (high mortality) were not statistically significant (*p* = 0.12). (Table 4)

**Table 4:**
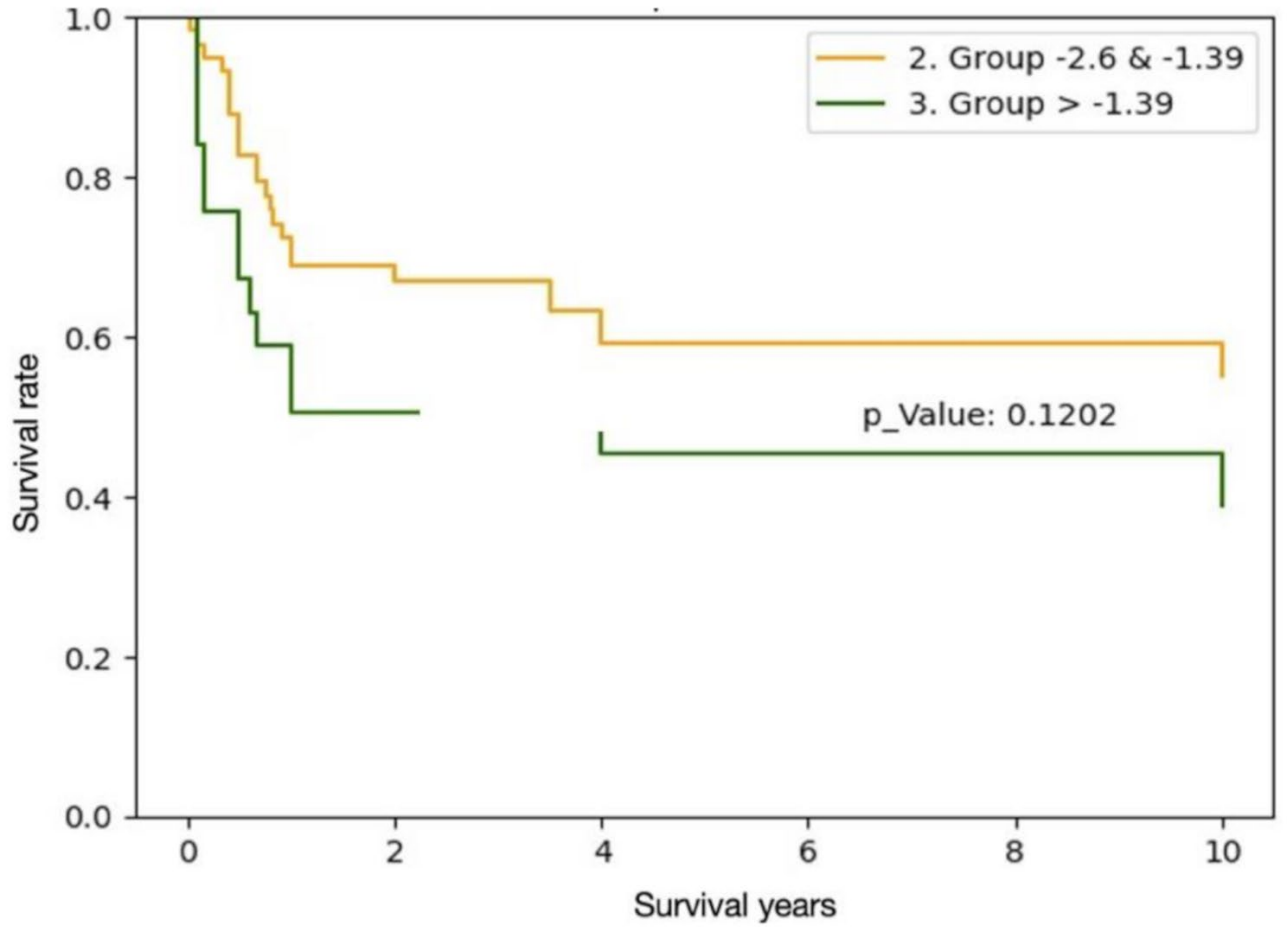
Kaplan-Meier survival curve based on preoperative ALBI scores

#### APRI scoring

Preoperative APRI scoring revealed that Group 1 (APRI < 0.43) showed the poorest prognosis graphically, contrary to expectations. However, log-rank analysis showed no statistically significant differences in 10-year survival among the groups (*p* = 0.17). The small sample size of Group 1 (*n* = 7) and confounding factors (four cases with high CMV DNA replication) likely contributed to this finding. (Table 5)

**Table 5:**
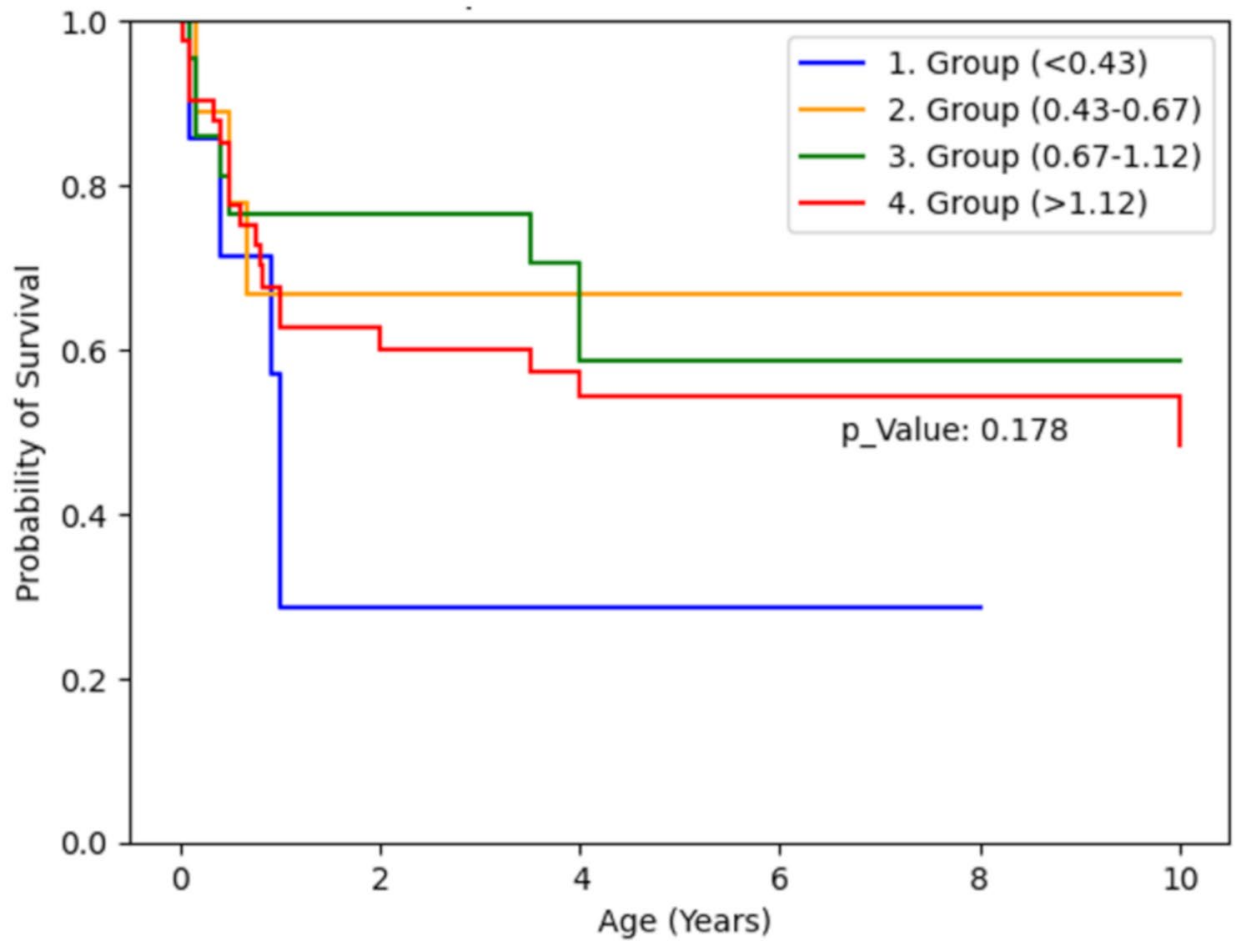
Kaplan-Meier survival curve based on preoperative APRI scores

#### Postoperative APRI scoring

At one month postoperatively, APRI scores did not significantly affect survival (*p* = 0.455). However, by the third month, Group 4 patients (APRI > 1.12) demonstrated significantly lower 10-year survival rates (*p* = 0.00094). Linear regression analysis indicated a trend of increasing APRI scores with older surgical ages, but irregular data distribution precluded statistical significance. (Table 6)

**Table 6:**
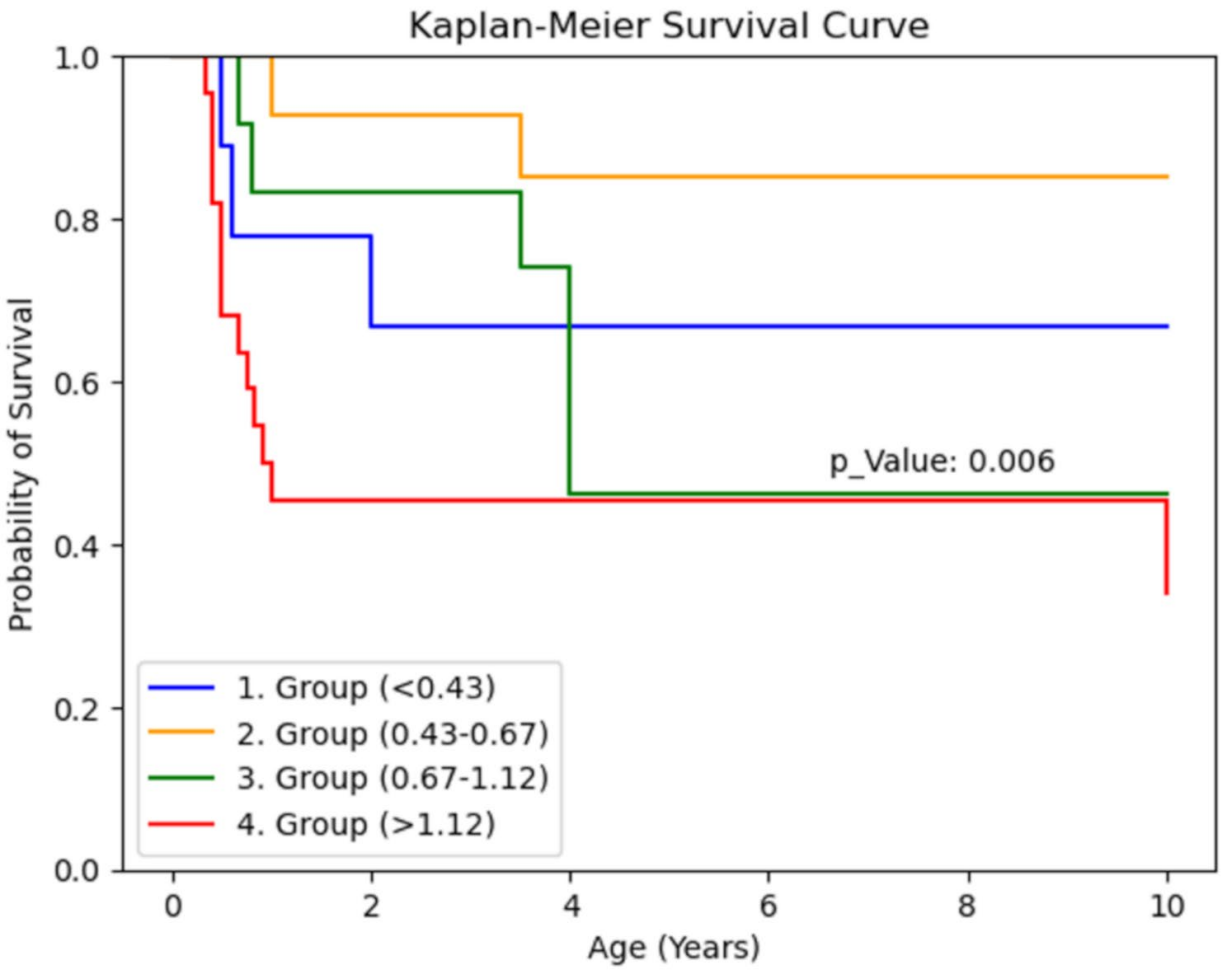
Kaplan-Meier survival curve based on Post-operative APRI scores

#### METAVIR scoring

Patients with METAVIR scores of 4 demonstrated poorer 10-year survival graphically, but the differences were not statistically significant (*p* = 0.26). A comparison of isolated biliary atresia cases and those with associated anomalies showed worse 10-year survival for cases with anomalies, though the small sample size (*n* = 3) led to low statistical significance (*p* = 0.171). The 10-year survival rate for cases without anomalies was 69.6%. (Table 7)

**Table 7:**
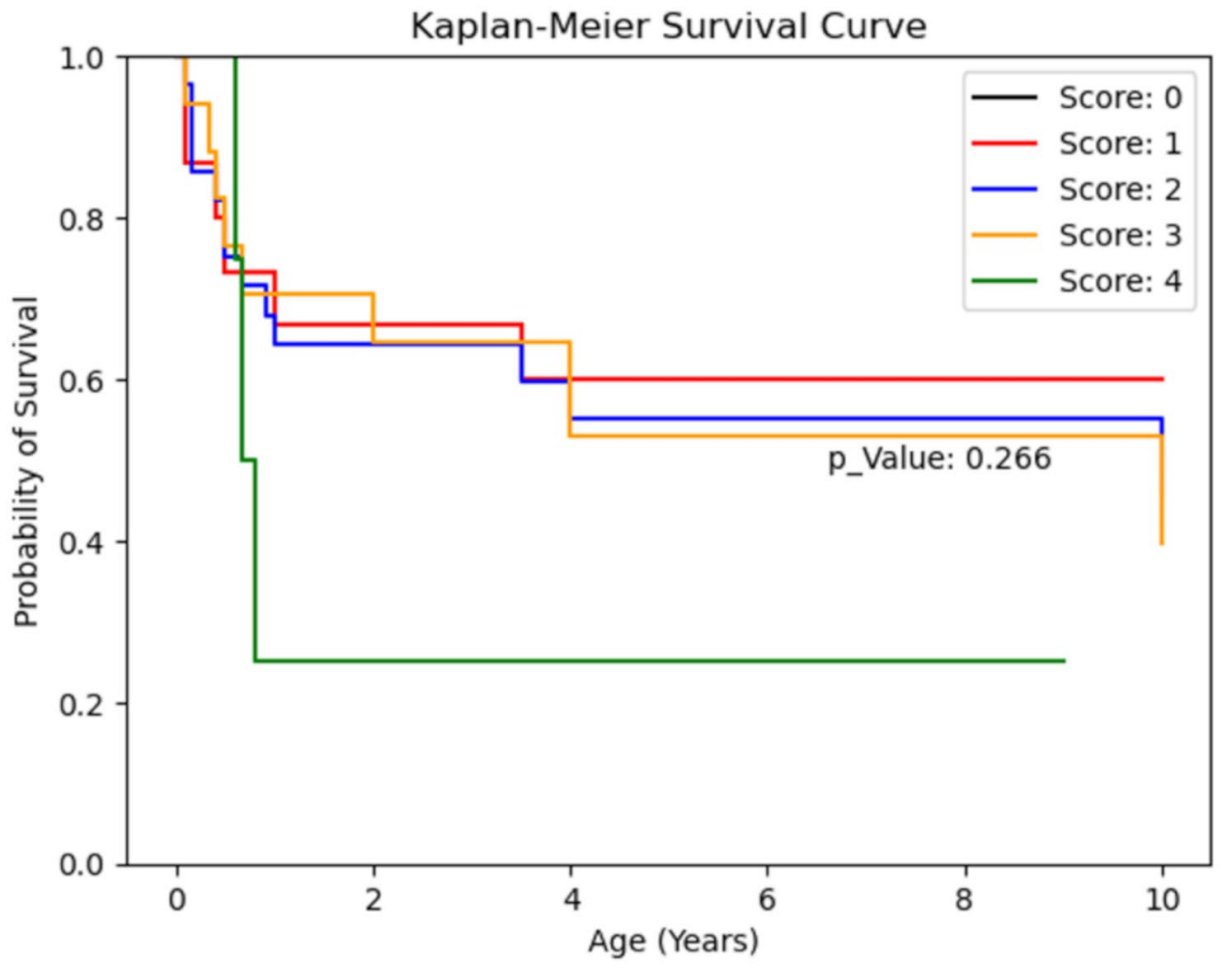
Kaplan-Meier survival curve based on postoperative METAVIR scores

### Laboratory values

Median laboratory values showed improvement from preoperative to postoperative status:

- Preoperative total bilirubin: 8.59 mg/dL → Postoperative: 1.92 mg/dL.
- Preoperative APRI: 1.135 → Postoperative: 0.81.

These findings suggest overall clinical improvement and a phase of liver regeneration in the postoperative period for the majority of patients.

## Discussion

### Clinical relevance of scoring systems in biliary Atresia

#### ALBI score and its applications

The ALBI score was initially developed to evaluate liver function in patients with hepatocellular carcinoma (HCC), functioning independently of underlying fibrosis. It has since demonstrated prognostic value across various liver diseases, including hepatitis B and C-related cirrhosis, alcoholic liver disease, and primary biliary cholangitis. Its unique strength lies in using only two objective laboratory parameters—albumin and bilirubin—which are central to the pathophysiology of liver disease. Unlike other prognostic tools like Child-Pugh scoring that incorporate subjective assessments, ALBI offers an objective reflection of liver reserve. However, our study did not demonstrate a significant role for the ALBI score in predicting prognosis among biliary atresia (BA) patients. This finding suggests that the mechanisms driving hepatic dysfunction in BA may differ substantially from those in the adult diseases for which ALBI was originally validated.

Although the ALBI score was originally developed to assess liver function in hepatocellular carcinoma, its utility in pediatric populations, particularly in biliary atresia, remains unclear. In our study, we included preoperative ALBI scores with the hypothesis that the score—based on albumin synthesis and bilirubin clearance—might reflect hepatic reserve in the setting of cholestatic injury. Despite this theoretical relevance, both preoperative and postoperative ALBI scores failed to demonstrate significant prognostic value. While postoperative ALBI was also calculated, the lack of statistical significance led us to exclude it from the final analysis. Our findings suggest that ALBI, although attractive due to its simplicity and objectivity, may not capture the complex regenerative and fibrotic dynamics unique to infantile cholangiopathies such as biliary atresia.

#### APRI score as a prognostic tool

The APRI score has been established as a non-invasive marker of liver fibrosis in several chronic liver diseases. Muntean et al. recently validated the utility of APRI specifically in paediatric liver disease, finding a significant correlation with outcomes in patients with biliary atresia [13]. Our study found that preoperative APRI scores did not have a significant impact on prognosis (*p* = 0.17), which is in contrast to some of the existing literature.

Interestingly, while the literature suggests that cases categorised in group 1 (APRI < 0.43) typically have the most favourable prognosis, our small sample size in this group (*n* = 7), coupled with confounding factors (high CMV replication in four cases), likely precluded statistical significance. However, the post-operative APRI score at 3 months proved to be a valuable prognostic indicator, with patients in group 4 (APRI > 1.12) showing significantly worse outcomes (*p* = 0.00094). This finding underscores the importance of monitoring APRI scores post-surgery, as they may provide critical insights into patient recovery and long-term outcomes following biliary atresia interventions. Based on this finding, we recommend that patients with 3-month postoperative APRI scores > 1.12 should receive more intensive monitoring and earlier consideration for transplantation listing.

#### Limitations of APRI

One of the limitations of our study lies in the inherent complexity of the pathophysiology of biliary atresia. While the APRI score has been previously validated as a marker of liver fibrosis and used in the context of biliary atresia, it primarily reflects static fibrosis rather than the dynamic changes seen post-Kasai portoenterostomy (KPE). Biliary atresia is characterized by rapidly progressive cholangiopathy and fibrosis prior to KPE, followed by a potential regenerative phase once biliary drainage is reestablished. We hypothesized that the pre-existing fibrosis, the rate of fibrosis progression or regression post-KPE, and the regenerative capacity of the liver together may influence long-term prognosis. However, relying solely on preoperative parameters to predict outcomes in such a dynamic disease is inherently challenging.

Moreover, the postoperative period is affected by multiple variables—such as infection, cholangitis, immune response, and nutritional status—making it difficult for a single score to comprehensively reflect prognosis. In our study, we observed considerable variability in APRI scores postoperatively, with a general trend toward improvement. This decline may indicate actual histological regression of fibrosis, although our retrospective design and lack of sequential biopsies limit the ability to confirm this histologically. Nevertheless, the trend of decreasing APRI scores in patients with favorable outcomes supports the notion that APRI may reflect regenerative changes post-KPE.

#### Limitations of METAVIR and other histological scoring systems

The METAVIR score is a semiquantitative histopathological grading system widely used to evaluate liver fibrosis. Its limitations include sampling variability, inter-observer discrepancies, and the static nature of the assessment. In our study, while patients with METAVIR scores of 4 demonstrated poorer survival graphically, these differences were not statistically significant. This finding highlights an important distinction between pediatric and adult liver fibrosis. The potential for reversible recovery from liver fibrosis in neonates and infants likely differs from adults, contributing to the limited prognostic utility of preoperative histological assessment in BA. This underscores the need for more dynamic, BA-specific prognostic tools that account for the regenerative capacity unique to the pediatric liver.

### Biological mechanisms of fibrosis and regeneration in neonatal liver

The literature highlights several biological mechanisms that may explain the distinct nature of liver fibrosis and regeneration in neonates compared to adults [14]. Oval cells derived from biliary epithelial cells, intrahepatic bile duct cilia (IHBCs), and liver progenitor-like cells (LPLCs) play critical roles in periportal injury repair following cholestasis [15]. Additionally, hepatocyte transdifferentiation into biliary epithelium via TGF-β signaling pathways has been documented [16]. The heightened telomerase activity and enhanced regenerative capacity observed during the neonatal and infant periods may explain why static scoring systems like METAVIR have limited prognostic utility in these populations [17]. This biological underpinning helps explain why some infants with severe preoperative fibrosis may still achieve favorable outcomes following Kasai portoenterostomy.

### Age at operation and outcomes

Our analysis revealed an unexpected finding regarding age at operation, with patients operated on before 30 days of age showing lower 10-year NLS rates compared to other groups. This finding should be interpreted with caution due to the small sample size in this subgroup. Only one patient in this early-surgery cohort had preoperative CMV positivity, and we did not identify specific comorbidities distinguishing these cases from others. While it is tempting to speculate that these infants might represent a distinct disease phenotype with earlier prenatal onset of biliary ductal obliteration, we lack embryological and molecular evidence to support this hypothesis. The results may represent a statistical anomaly due to the limited sample size rather than reflecting true biological differences. Our finding contradicts the established principle that earlier intervention generally yields better outcomes in biliary atresia, suggesting that additional factors beyond surgical timing influence long-term prognosis in these youngest patients.

### Temporal trends and Long-term outcomes

Our analysis demonstrated a statistically significant improvement in patient outcomes across three decades of experience, encompassing 96 cases. The most dramatic improvement was observed between the earliest (1990–2000, *n* = 18) and most recent (2011–2022, *n* = 47) eras, with NLS rates increasing from 42.1 to 64.8% (*p* = 0.0029) and OS rates rising from 56.3 to 85.2% (*p* < 0.0001). This remarkable progress can be attributed to several key factors:

- Refinements in surgical techniques and expertise.
- Enhanced perioperative care protocols.
- Improved postoperative management strategies.
- Better infection control measures.

The intermediate era (2001–2010, *n* = 31) represented a transitional period, showing incremental improvements in both NLS and OS rates. While the improvements between consecutive eras were more gradual, the overall trend demonstrates consistent progress in surgical and medical management. The most recent era (2011–2022) showed particularly promising results, with OS rates exceeding 85%, highlighting the cumulative benefit of decades of experience and medical advances. The increasing number of cases per era (from 18 in 1990–2000 to 47 in 2011–2022) reflects the growing role of our center as a reference institution for biliary atresia management. This increased case volume likely contributed to improved outcomes through enhanced surgical experience and standardization of care protocols.

### Clearance of jaundice as a key outcome measure

Our study found that clearance of jaundice, defined as total bilirubin levels < 2 mg/dL and passage of colored stools within three months, was achieved in 30 cases (31.2%). This rate appears lower than some international series but is consistent with our overall native liver survival rates. The median APRI score for jaundice-free cases was 0.60, indicating significant improvement compared to preoperative values. Jaundice clearance serves as an important early indicator of successful bile drainage and correlates strongly with long-term native liver survival. This reinforces the importance of this parameter as a key outcome measure in future biliary atresia studies.

### Comparative international outcomes

The overall native liver survival and total survival rates in our series are comparable to those reported in large international registries and multicenter studies (Table 8). Our 10-year native liver survival rate of 52.4% aligns with data from Japan (52–62%) [24], while our overall survival rate of 69.6% is comparable to rates reported in the Netherlands (73%) [21] but lower than more recent series from the UK (87%) [18] and France (92%) [19].

**Table 8:** International data available on the outcomes of biliary Atresia

	Period	*N*:	Average Age of KPE (Days)	Clearence of Jaundice (6 mo)	4–5 Years of Native Liver Survival	4–5 Years of True Survival
UK & Wales [18]	1999–2009	443	54	56%	46%	90%
France [19]	2003–2009	329	59	33–39%	33–39%	87–92%
Switzerland [20]	1994–2004	48	59	N/a	37%	92%
Netherlands [21]	1987–2008	214	59,5	N/a	46%	73%
Canada [22]	1996–2002	150	55	N/a	39%	82%
USA [23]	1997–2000	104	61	40%	N/a	91%
Japan [24]	1989–1998	1381	65	57–62%	52–62%	70–78%
*Current Study*	*1990–2022*	*96*	*57*	*31,2% (3 mo)*	*56,8%*	*69,6%*

### Innovation and clinical implications

While both ALBI and APRI scores have been previously investigated in various liver diseases, our study’s innovation lies in the comprehensive assessment of their utility, specifically in biliary atresia, particularly identifying postoperative APRI at 3 months as a significant predictor of long-term outcomes. The finding that cases with an APRI score > 1.12 at 3 months postoperatively have significantly worse outcomes provides clinicians with a valuable tool for early risk stratification. This may guide more intensive monitoring and earlier consideration for transplantation in high-risk patients, potentially improving overall outcomes.

## Conclusion

Kasai portoenterostomy remains the first-line treatment for biliary atresia, with our study confirming that approximately half of patients can achieve 10-year survival with their native liver. Postoperative APRI score > 1.12 at 3 months emerged as a strong predictor of poor outcomes, while preoperative scoring systems showed limited predictive value. We observed significant improvements in both native liver survival and overall survival across three decades, highlighting the value of accumulated institutional experience and improved clinical protocols in managing this challenging disease.

## Data Availability

The datasets used and/or analyzed during the current study are available from the corresponding author on reasonable request.

## References

[1] ​Johnson PJ, Berhane S, Kagebayashi C, Satomura S, Teng M, Reeves HL, et al. Assessment of liver function in patients with hepatocellular carcinoma: A new Evidence-Based Approach—The ALBI grade. J Clin Oncol. 2015;33(6):550–8. 25512453 10.1200/JCO.2014.57.9151 PMC4322258

[2] ​Chan AWH, Chan RCK, Wong GLH, Wong VWS, Choi PCL, Chan HLY, et al. New simple prognostic score for primary biliary cirrhosis: Albumin-bilirubin score. J Gastroenterol Hepatol. 2015;30(9):1391–6. 25753927 10.1111/jgh.12938

[3] ​Wai C. A simple noninvasive index can predict both significant fibrosis and cirrhosis in patients with chronic hepatitis C. Hepatology. 2003;38(2):518–26. 12883497 10.1053/jhep.2003.50346

[4] ​ Suominen JS, Lampela H, Heikkilä P, Lohi J, Jalanko H, Pakarinen MP. APRi predicts native liver survival by reflecting portal fibrogenesis and hepatic neovascularization at the time of portoenterostomy in biliary Atresia. J Pediatr Surg. 2015;50(9):1528–31. 25783319 10.1016/j.jpedsurg.2014.11.046

[5] ​ Grieve A, Makin E, Davenport M. Aspartate Aminotransferase-to-Platelet ratio index (APRi) in infants with biliary atresia: prognostic value at presentation. J Pediatr Surg. 2013;48(4):789–95. 23583135 10.1016/j.jpedsurg.2012.10.010

[6] ​ Bedossa P, Poynard T. An algorithm for the grading of activity in chronic hepatitis C. Hepatology. 1996;24(2):289–93. 8690394 10.1002/hep.510240201

[7] ​Shen Q-L, Chen Y-J, Wang Z-M, Zhang T-C, Pang W-B, Shu J, et al. Assessment of liver fibrosis by fibroscan as compared to liver biopsy in biliary Atresia. World J Gastroenterol. 2015;21(22):6931–6. 26078570 10.3748/wjg.v21.i22.6931 PMC4462734

[8] ​Brumbaugh D, Mack C. Conjugated hyperbilirubinemia in children. Pediatr Rev. 2012;33(7):291–302. 22753787 10.1542/pir.33-7-291

[9] ​ Davenport M, Puricelli V, Farrant P, Hadzic N, Mieli-Vergani G, Portmann B, et al. The outcome of the older (≥ 100 days) infant with biliary Atresia. J Pediatr Surg. 2004;39(4):575–81. 15065031 10.1016/j.jpedsurg.2003.12.014

[10] ​Deguchi E, Yanagihara J, Iwai N. Bile duct patterns in the hilar region of the liver in two cases of biliary Atresia. J Pediatr Surg. 1990;25(3):307–10. 2313498 10.1016/0022-3468(90)90073-i

[11] Senyüz OF, Yeşildağ E, Emir H, Tekant G, Bozkurt P, Sarimurat N, Söylet Y. Diagnostic laparoscopy in prolonged jaundice. J Pediatr Surg. 2001;36(3):463–5. 10.1053/jpsu.2001.21621 11226997 10.1053/jpsu.2001.21621

[12] Redkar R, Karkera PJ, Raj V, Bangar A, Hathiramani V, Krishnan J. Outcome of biliary Atresia after kasai’s portoenterostomy: A 15-year experience. Indian Pediatr. 2017;54(4):291–4. 10.1007/s13312-017-1091-5. Epub 2017 Feb 2. PMID: 28159941. 28159941 10.1007/s13312-017-1091-5

[13] Muntean A, Kronfli R, Makin E, Davenport M. The AST-to-Platelet ratio index (APRi) at Kasai portoenterostomy: standing the test of time. J Pediatr Surg. 2023;58(12):2347–51. 10.1016/j.jpedsurg.2023.06.012. Epub 2023 Jun 28. PMID: 37468346. 37468346 10.1016/j.jpedsurg.2023.06.012

[14] Wells RG. April. M.D.^*,1^. Hepatic fibrosis in children and adults. Clinical Liver Disease. 2017;9(4):99–101.| 10.1002/cld.623 10.1002/cld.623 PMC6467142 30992969

[15] Philip N, Newsome. M, Hussain. A, Theise ND. Hepatic oval cells: helping redefine a paradigm in stem cell biology. Curr Top Dev Biol. 2004;61:1–28. 15350395 10.1016/S0070-2153(04)61001-5

[16] Johanna R, Kari S, Huppert A, Simone N, Kurial T, Bernadette Y, Hsu., Ashley C, Bryan D, Rebekah K, Feng C, Milad R, Hubert Y, Aras L, Mattis N, Anne-Laure R, Philip J, Rosenthal., Stacey S, Stacey H. S., Huppert., holger, willenbring. (2018). De Novo formation of the biliary system by TGFβ-mediated hepatocyte transdifferentiation. Nature, 557(7704):247–51. 10.1038/s41586-018-0075-5 PMC6597492 29720662

[17] Eva S, Lola M, Reid. Human telomerase activity, telomerase and telomeric template expression in hepatic stem cells and in livers from fetal and postnatal donors. Eur J Gastroenterol Hepatol. 2009;21(10):1191–8. 19240645 10.1097/MEG.0b013e32832973fc PMC2743773

[18] Davenport M, Ong E, Sharif K, Alizai N, McClean P, Hadzic N, Kelly DA. Biliary Atresia in England and wales: results of centralization and new benchmark. J Pediatr Surg. 2011;46(9):1689–94. 21929975 10.1016/j.jpedsurg.2011.04.013

[19] Chardot C, Buet C, Serinet MO, Golmard JL, Lachaux A, Roquelaure B, Gottrand F, Broué P, Dabadie A, Gauthier F, Jacquemin E. Improving outcomes of biliary atresia: French National series 1986–2009. J Hepatol. 2013;58(6):1209–17. 23402746 10.1016/j.jhep.2013.01.040

[20] Wildhaber BE, Majno P, Mayr J, Zachariou Z, Hohlfeld J, Schwoebel M, Kistler W, Meuli M, Le Coultre C, Mentha G, Belli D, Chardot C. Biliary atresia: Swiss National study, 1994–2004. J Pediatr Gastroenterol Nutr. 2008;46(3):299–307. 18376248 10.1097/MPG.0b013e3181633562

[21] De Vries W, Homan-Van der Veen J, Hulscher JB, et al. Twenty-year transplant-free survival rate among patients with biliary Atresia. Clin Gastroenterol Hepatol. 2011;9:1086–91. 21820397 10.1016/j.cgh.2011.07.024

[22] Schreiber RA, Barker CC, Roberts EA, Martin SR, Alvarez F, Smith L, Butzner JD, Wrobel I, Mack D, Moroz S, Rashid M, Persad R, Levesque D, Brill H, Bruce G, Critch J. Canadian pediatric hepatology research group. Biliary atresia: the Canadian experience. J Pediatr. 2007;151(6):659–65. 665.e1. 18035148 10.1016/j.jpeds.2007.05.051

[23] Shneider BL, Brown MB, Haber B, et al. A multicenter study of the outcome of biliary Atresia in the united states, 1997 to 2000. J Pediatr. 2006;148:467–74. 16647406 10.1016/j.jpeds.2005.12.054

[24] Nio M, Ohi R, Miyano T, Saeki M, Shiraki K, Tanaka K. Japanese biliary Atresia registry. Five- and 10-year survival rates after surgery for biliary atresia: a report from the Japanese biliary Atresia registry. J Pediatr Surg. 2003;38(7):997–1000. 12861525 10.1016/s0022-3468(03)00178-7

